# Impact of Vector Dispersal and Host-Plant Fidelity on the Dissemination of an Emerging Plant Pathogen

**DOI:** 10.1371/journal.pone.0051809

**Published:** 2012-12-19

**Authors:** Jes Johannesen, Xavier Foissac, Patrik Kehrli, Michael Maixner

**Affiliations:** 1 Institute of Zoology, University of Mainz, Mainz, Germany; 2 INRA, UMR1332 Fruit Biology and Pathology, Villenave d'Ornon, France; 3 Université Bordeaux Ségalen, UMR1332 Fruit Biology and Pathology, Villenave d'Ornon, France; 4 Station de recherche Agroscope Changins-Wädenswil ACW, Protection végétaux, Nyon, Switzerland; 5 Julius Kühn-Institute, Institute for Plant Protection in Fruit Crops and Viticulture, Bernkastel-Kues, Germany; University of Utah, United States of America

## Abstract

Dissemination of vector-transmitted pathogens depend on the survival and dispersal of the vector and the vector's ability to transmit the pathogen, while the host range of vector and pathogen determine the breath of transmission possibilities. In this study, we address how the interaction between dispersal and plant fidelities of a pathogen (stolbur phytoplasma tuf-a) and its vector (*Hyalesthes obsoletus*: Cixiidae) affect the emergence of the pathogen. Using genetic markers, we analysed the geographic origin and range expansion of both organisms in Western Europe and, specifically, whether the pathogen's dissemination in the northern range is caused by resident vectors widening their host-plant use from field bindweed to stinging nettle, and subsequent host specialisation. We found evidence for common origins of pathogen and vector south of the European Alps. Genetic patterns in vector populations show signals of secondary range expansion in Western Europe leading to dissemination of tuf-a pathogens, which might be newly acquired and of hybrid origin. Hence, the emergence of stolbur tuf-a in the northern range was explained by secondary immigration of vectors carrying stinging nettle-specialised tuf-a, not by widening the host-plant spectrum of resident vectors with pathogen transmission from field bindweed to stinging nettle nor by primary co-migration from the resident vector's historical area of origin. The introduction of tuf-a to stinging nettle in the northern range was therefore independent of vector's host-plant specialisation but the rapid pathogen dissemination depended on the vector's host shift, whereas the general dissemination elsewhere was linked to plant specialisation of the pathogen but not of the vector.

## Introduction

The emergence of vector-transmitted plant symbionts/pathogens depends highly on vector dispersal and the host specificity of vector and pathogen [1]. For pathogens to emerge to epidemic levels, vector transmission to compatible host populations is required as are frequent encounters between vector and pathogen within a suitable environment [2]. While vector dispersal is essential for pathogens to encounter new host individuals and spread disease, vector feeding-behaviour (monophagous vs. polyphagous) can in distinct ways determine the pathogen's end host and the breadth of transmission potential [3]. Narrow host ranges may lead to specialised transmission cycles whereas polyphagous vectors have the potential to expose many host species to pathogens and different pathogens to specific hosts [4]–[6]. Broad feeding ranges may result in transmission to suboptimal or non-reservoir hosts with unsynchronised host-pathogen syndromes being the consequence [2], [5], [7]. These so-called spillover events, although having serious impacts on the infected non-reservoir hosts, are less important for pathogen dissemination, which is determined by the interaction between vector and pathogen and their common natural hosts. The incidence of these events has been related to relative host densities [8] and geographic range expansion of the vector [9], [10].

Phytoplasma are wall-less, non-helical prokaryotes that colonise plant phloem and depend of phloem-feeding insect vectors (leafhoppers, planthoppers, and psyllids) for transmission [11]. Phytoplasma are important plant pathogens that are known to induce disease in several hundred plant species worldwide, including major agricultural crops, ornamental plants and timber trees [reviewed in 3]. Phytoplasma of the stolbur (16Sr-XIIA) group [12] (proposed name: *Candidatus Phytoplasma solani* [13]) cause yellows diseases with high economic loss in grapevine (bois noir), maize (maize redness), lavender and various Solanaceous crops in Europe. Stolbur phytoplasmas have obligate vector-based transmission and stolbur diseases are highly or exclusively determined by pathogen and vector reservoirs in weedy host plants [14]–[19]. Stolbur phytoplasma has two main genetic clusters, tuf-a and tuf-b, characterised by a diagnostic SNP in the tuf-gene [16]. While tuf-a so far has been associated predominately with stinging nettle (*Urtica dioica*) [16], [20], tuf-b is found in a range of other weedy plants [17], field bindweed (*Convolvolus arvensis*) being the dominant reservoir plant throughout most of Europe [21].

The main, and in most regions only known, vector of stolbur phytoplasma is the planthopper *Hyalesthes obsoletus* Signoret, 1865 (Hemiptera: Cixiidae) [22]–[24]. Principal natural hosts of the vector correspond to the reservoir plants of stolbur, field bindweed again being the dominant host in most parts of Europe [21] but with stinging nettle being regarded as the natural host plant in northern Italy [25]. In the economically important grapevine disease bois noir, the epidemiology is coupled to the infection of these two herbaceous host plants and not to grapevine, since grapevine is not a nymphal substrate for *H. obsoletus* and, consequently, a dead-end host for stolbur. Vectors infected with stolbur nearly always (>95%) have stolbur type of the host plant from which they were collected [26].

The vector is predominantly Mediterranean. The northern distribution range limit is xerothermic habitats in Germany and northern France (Alsace) [27], mainly in vineyards on slopes of river valleys. Until about 25 years ago, the vector was rare in this area [28] and associated mainly with bindweed. Today, the vector's rapid population growth coincides with a host range expansion to stinging nettle, although an increase of vector populations on field bindweed, the traditional host, is also observed. The host shift to stinging nettle in the northern range has been accompanied by the emergence of tuf-a, which is now the dominant stolbur strain in most North-western European wine-growing regions [29]–[31].

**Figure 1:**
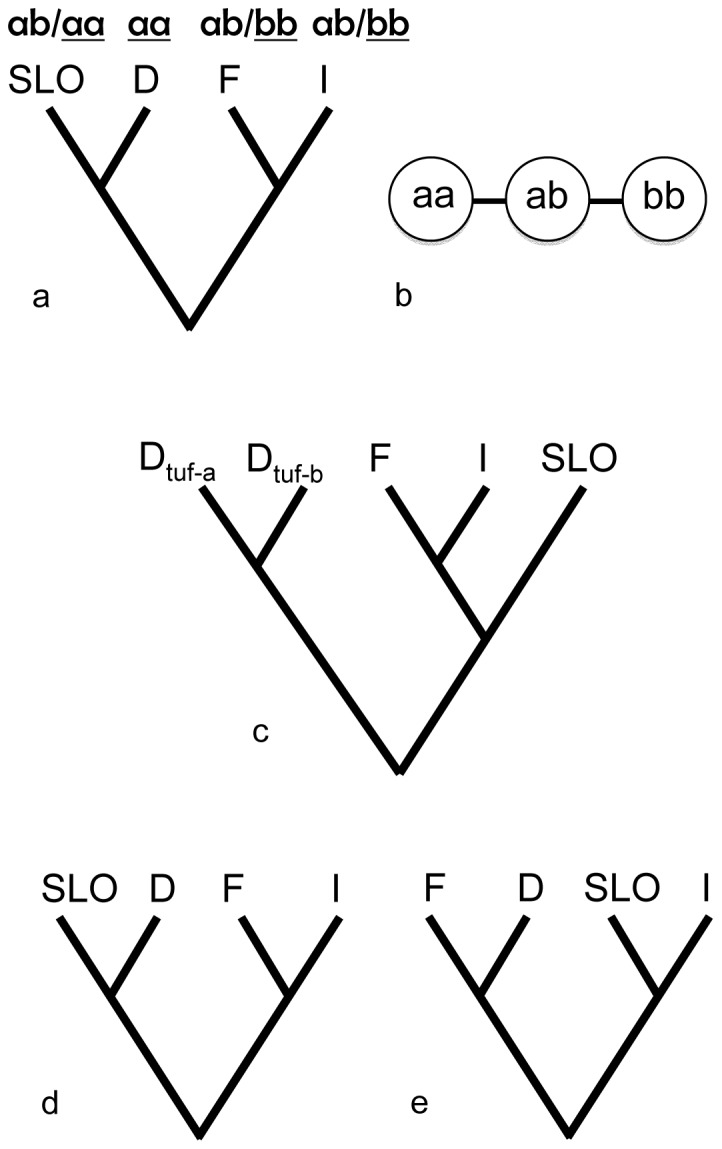
Schematic presentation of historical dispersal of *H. obsoletus* and expected co-dispersal scenarios of *H. obsoletus* and tuf-a stolbur. (a) Historical dispersal of *H. obsoletus* showing origin of German (D) *H. obsoletus* in Slovenia (SLO) inferred from (b) genealogical relationships of haplotypes aa, ab and bb. The haplotype aa evolved east of the Italian-Slovenian karst divide. Italian (I) and French (F) *H. obsoletus* exhibit the derived haplotype bb, which is found west of the divide only. The ancestral haplotype ab is found throughout southern Europe but not in Germany [32]. (c) Scenario 1: Stolbur tuf-a transferred from field bindweed to stinging nettle in the course of *H. obsoletus*' host shift. (d) Scenario 2: expected phylogeny of monophyletic tuf-a stolbur assuming historical co-dispersal of *H. obsoletus* and tuf-a stolbur from Slovenia into Germany. (e) Scenario 3: dispersal of monophyletic tuf-a stolbur from France into Germany that is independent of host race formation of *H. obsoletus* in Germany. An Italian ancestry is assumed in all scenarios.

The northern populations of *H. obsoletus* from both host plants are fixed for the mtDNA haplotype “aa” [32] but are genetically divergent at microsatellite loci [33] (M. Imo, M. Maixner & J. Johannesen, unpublished data). This indicates that vector populations using stinging nettle in this area originated from local bindweed populations and that two genetic host-race populations have evolved perhaps since the host shift over the past 25 years (Fig. 1a,b). Haplotype “aa” has a historical origin east of the Italian-Slovenian karst divide and reached Germany and Alsace in a westward migration north of the European Alps. However, a northwards migration west of the Alps via France of vectors carrying the haplotype, “bb”, has reached the southernmost Germany and Alsace, establishing a secondary contact zone. Both haplotypes belong to a western European genealogical lineage that originated south of the European Alps and spread in a post-glacial circum Alpine expansion [32].

The parallel associations among stolbur strains, vector populations and host plants in Germany and Alsace imply two independent vector transmission cycles of stolbur phytoplasma, one associated with stinging nettle as a new plant host and one associated predominately with field bindweed but also involving other host plants [16]. In contrast to the situation in Germany and Alsace, ancestral Mediterranean *H. obsoletus* populations associated with stinging nettle and field bindweed cannot be differentiated genetically at microsatellite loci (M. Imo, M. Maixner & J. Johannesen, unpublished data). The lack of plant specificity in *H. obsoletus* in southern countries thus contrasts the apparent global host specificity of stolbur strains.

The geographic range expansion of *H. obsoletus* coupled to an extant host-shift with specialised populations in a newly colonised area, and the acquisition and dissemination of a new obligate vectored pathogen-strain in this area offers the unique opportunity to study the interaction between geographic range expansion and host shift (plant fidelity) of a vector on the dissemination of an obligate vectored plant pathogen. In this study, we analyse the origin and dissemination of stinging nettle associated tuf-a stolbur in Western Europe where this strain has emerged as a major pathogen of grapevine. Specifically, we analyse whether the rapid dissemination of the pathogen in the novel geographic range, Germany and Alsace, is related to the vector's host-plant shift or influenced by geographic range expansion. We test three scenarios: 1) Stolbur was transferred from field bindweed to stinging nettle in the course of the vector's host shift between these two plants (Fig. 1c). In this case, monophyly of stolbur infecting field bindweed to stinging nettle in the geographic region of the host shift relative to regions outside is expected. Because tuf-a and tuf-b stolbur phytoplasma have been related to stinging nettle and field bindweed, respectively, on a European scale we do not expect this transfer to have happened. However, the transfer has so far not been subjected to a quantitative analysis in the area of the host shift. 2) Tuf-a stolbur co-migrated with resident, i.e. mtDNA haplotype “aa”, vectors into Germany (Fig. 1d). 3) The introduction of tuf-a stolbur is linked to the secondary contact between northwards emigrating “bb” vectors from France and resident “aa” vectors (Fig. 1e). In the second scenario, German tuf-a stolbur should be most related to Slovenian strains whereas the third scenario is supported if French and German tuf-a strains are the most related and these strains are associated with *H. obsoletus* of the “bb” haplotype. If scenario 2 is supported it suggests that stinging nettle intermediately may have been lost as a host, perhaps with stolbur tuf-a relying on an alternative vector. Scenario 3 implies that the dissemination on stinging nettle in Germany is caused by a new introduction of stolbur tuf-a. In the third scenario, the vector's host-shift is independent of stolbur infection but essential for the dissemination in a new environment.

## Materials and Methods

The analyses were based on frequency distributions and genealogies of four stolbur genes and two *H. obsoletus* mtDNA genes. Stolbur gene sequences were obtained by direct sequencing of genomic DNA isolated from infected *H. obsoletus* collected on stinging nettle or field bindweed. Stolbur tuf-a was investigated from the circum Alpine geographic expansion range of *H. obsoletus* (Tables 1 and 2, Appendix S1, S2) and based on collections made between 2005 and 2010.

**Table 1:** Sample locations of tuf-a and -b strains sequenced at four genes, *stamp*, *stol-11*, *secY* and *vmp1*.

					Number of sequences		
Stolbur	Country	Location	Region	*stamp*	*stol-11*	*secY*	*vmp1*
tuf-a	Germany	Lieser	Mosel	3	4	3	4
		Bacharach	Rhine	1	3	3	2
		Ungstein	Pfalz	2	2	2	2
		Neuweier	Baden	2	2	2	2
		Weinsberg	Württemberg	2	2	2	2
		Randersacker	Franken	2	2	1	1
	Switzerland,	Arlesheim	Basel	0	1	1	1
	north of Alps	Hallau	Schaffhausen	1	4	3	4
		Eglisau	Zürich	0	1	1	1
		Bellerive	Murtensee	1	2	2	1
		La Landeron	Bielersee	1	4	5	3
		Espesse	Vaud	1	3	3	4
		Russin	Geneve	1	2	2	2
	Italy*	Rome	Lazio	2	3	3	2
		Pisa	Toscana	1	2	2	1
		Verona	Venetio	2	0	3	3
		Eisacktal	Trentino	2	2	2	1
		Cembratal	Südtirol/Alto Adige	4	6	6	2
		Gudo	Ticino	0	1	1	1
		Serentina	Ticino	0	2	2	0
		Biasca	Ticino	2	2	2	2
	France	Bretenieres	Bourgogne	1	1	1	1
		Charentes	Charentes	0	1	1	1
	Slovenia	Osevljek	Osijek-Baranja	5	4	5	6
	& Croatia	Nova Gorica	Goriška	2	2	2	2
		No information	-	1	1	1	1
		Zeljezna Gora	Međimurje	2	2	1	2
tuf-b	Germany	Lieser	Mosel	1	1	1	1
		Bacharach	Rhine	1	1	1	1
		Boppard	Rhine	1	1	1	1
		Neuweier	Baden	1	1	1	1
		Norheim	Nahe	1	1	1	1
	Italy	Eisacktal	Trentino	1	1	1	1
		Reggio	Emilia-Rom.	1	1	1	1
	France	Beaune	Côte d'Or	0	1	1	1
	Slovenia	Nova Gorica	Goriška	1	1	1	1

The b-strains were used for outgroup comparisons. Map locations are shown in Appendix S1 and S2.

* Includes the Swiss canton Ticino south of the Alps.

**Table 2:** Sample localities and mtDNA haplotypes for *Hyalesthes obsoletus* in the present study (A) and compared with summary data from [32] (B).

					Haplotype														
Study	Locality	Country	Plant	n	aa	ab	ad	af	aj	bb	cd	db	ib	ig	kb	lb	nb	ob	tb
A	Neuweier	D	U	3	3														
	Neuweier	D	C	3	3														
	Arlesheim	CH	U	3	1					1							1		
	Hallau	CH	U	6	2					2							2		
	Bellerive	CH	U	3						3									
	Le Landeron	CH	U	5						5									
	Le Landeron	CH	C	5						5									
	Espesses	CH	C	2						2									
	Morges	CH	U	5						5									
	Russin	CH	U	4						3						1			
	Charentes	F	C	5						5									
	Beaune	F	C	3						3									
	Savigny les Beaunes	F	C	3						3									
	Pupilin	F	U	2						2									
	Mesnay	F	U	1						1									
	Gevingey	F	U	1						1									
	Mévouillon	F	L	2		2													
	Felthurns	I	C	5						4					1				
	Cembra	I	U	3		2				1									
	Modena	I	U	5		1	1		1	2									
	Reggio	I	C	4						2								2	
	Rome	I	U	3		2							1						
	Osevljek	SLO	U	5		4													1
	*Total A*			*81*	*9*	*11*	*1*		*1*	*50*			*1*		*1*	*1*	*3*	*2*	*1*
B	Germany	D	U/C	34	31					3									
	France (Alsace)	F	U	5	5														
	France	F	U	9		3				5		1							
	Italy	I	U	22		12		4		2	1		2	1					
	Slovenia/Austria	SLO/A	U/C	24	13	11													
	*Grand total A+B*			*175*	*58*	*37*	*1*	*4*	*1*	*60*	*1*	*1*	*3*	*1*	*1*	*1*	*3*	*2*	*1*

Locations are shown in Fig. 1. The haplotype network of the sequences is presented in Appendix S9. Country: A, Austria, D, Germany; CH, Switzerland; F, France; I, Italy. Plant: C, Convolvulus arvensis; U, Urtica dioica; L, Lavandula angustifolia.

*H. obsoletus* was analysed for mtDNA sequences NADH dehydrogenase subunit I (400 bp), Cytochrome Oxidase subunit I (CO I) (198 bp), tRNA(Leu) (67 bp) and Cytochrome Oxidase subunit II (CO II) (523 bp), using the primers and protocol in [32]. New mtDNA sequences for the present study were obtained from the western area of tuf-a's putative origin, Italy and France, in the contact zone Switzerland, and at one south German location (Table 2). Samples from these areas west of the Alps were underrepresented in [32]. Individuals were collected between 2008 and 2010. We further sequenced the mtDNA from tuf-a positive *H. obsoletus* from Slovenia and Croatia. Thus, all stolbur tuf-a genotypes were related to a *H. obsoletus* mtDNA haplotype. The mtDNA distributions of the present study were combined with data from [32] to test demographic expansions of *H. obsoletus*. The haplotypes have the Genbank accession numbers: COII: EU155640–44, -48 and JX025159–63; ND1: EU155649–50, -52, 54–55 and JQ977743 [32]; this study].

The dispersal history of tuf-a stolbur was inferred from the genealogy and frequency distribution of partial gene sequences of two membrane proteins, *vmp1* [34], [35] and *stamp* [36] and two house-keeping genes, *stol-11* and *secY* [18], [37]. Stolbur infection in *H. obsoletus* was assessed with the stolbur specific primers *stol-11*: *STOL-11*f2: 5′-TAT-TTT-CCT-AAA-ATT-GAT-TGG-C-3′ and *STOL-11*r1: 5′-TGT-TTT-TGC-ACC-GTT-AAA-GC-3′ [37], *vmp1*: TYPH10F-AAC-GTT-CAT-CAA-CAA-TCA-GTC and TYPH10R 5′-CAC-TTC-TTT-CAG-GCA-ACT-TC-3 [18], *secY*: PosecF3 5′-GGA-TTG-ATA-GAT-GCT-GCC-CC-3′ and PosecR3 5′-GCC-CCT-ATA-ACG-GTG-ATT-TTG-A-3′ [18], *stamp*: stamp-F1 5′-TTC-TTT-AAA-CAC-ACC-AAG-AC-3′and stamp-R1 5′-AAG-CCA-GAA-TTT-AAT-CTA-CC-3′, using the published protocols [18], [36], [37].

DNA product amplification was performed in an end volume of 25 µl consisting of 1 µl forward and backward primer (10 pmol/µl), 1 µl DNA extract and 22 µl H_2_O sterile. Each sample was covered with 15 µl Chill Out 14 Liquid Wax (MJ Research). The PCR was performed with “Ready To Go™ PCR Beads” (0.5 ml tubes; Amersham Pharmacia Biotech) using a PTC-100 thermocycler (MJ Research).

Reference sequences of all stolbur genes were obtained by sequencing each gene in the sense and anti-sense direction. Hereafter, *secY*, *stol-11* and *stamp* were sequenced in one direction. *Vmp1*, having an amplification product of about 1500 bp, was sequenced in both directions.

### Data analysis

Sequence diversity of each of the four tuf-a genes was estimated within four pre-defined geographic regions, the host-shift population Germany (1), a putative transition population Switzerland (2), and the two putative regions of origin, Italy and southern France (3), and Slovenia and Croatia (4). Diversity was estimated as the number of sequences, nucleotide diversity and mean number of pair-wise differences between sequences using Arlequin 3.5 [38]. The number of synonymous and non-synonymous mutations was calculated with DnaSP v5.0 [39].

To test for monophyly of tuf-a genotypes, these were compared to nine strains of tuf-b from Germany (N = 5), Italy (N = 2), France (N = 1) and Slovenia (N = 1); all strains came from different sites (Table 1). Phylogenetic relationships among *secY* and *stol-11* gene sequences were analysed with haplotype networks, TCS 1.2.1 [40]), due to low variability among these sequences. For the polymorphic *vmp1* and *stamp* genes, we first determined the independence of of tuf-a and tuf-b (stinging nettle- and bindweed-associated) sequences with UPGMA phylogenetic analysis, as no appropriate out-group sequences were available.

After confirming monophyly of the stinging nettle associated sequences (see Results), the phylogenetic relationships of *vmp1* and *stamp* genotypes were analysed with Neighborjoining (NJ), Maximum Likelihood (ML), and Maximum Parsimony (MP) with tuf-b genotypes as outgroups. For NJ and ML we used the HKY substitution model without gamma distribution, found by the online software “Findmodel” (Los Alamos National Laboratory, http://www.hiv.lanl.gov/). For MP, all characters were weighted equally. Significance of branch lengths was estimated in heuristic searches with random addition of sequences and TBR branch swapping in 2000 bootstrap replicates. 50%-majority rule consensus trees based on bootstrap search were computed for all tree algorithms. All phylogenetic analyses were performed with PAUP version 4.08b for the Macintosh [41].

We tested neutral molecular evolution of tuf-a genotypes for the genes *vmp1*, *secY* and *stamp* using Tajima's D [42] (the number of unique mutations among all sequences relative to the total number of mutations) and Fu's Fs (the probability of having a number of haplotypes greater or equal to the observed number of samples drawn from a constant-sized population) [43] with Arlequin 3.5. Diversifying selection has been shown for *stamp* across tuf-a and -b genotypes [36] but was not tested within tuf-a, the objective of the present study. We tested for clock-like evolution of branch lengths in *vmp1* and *stamp* with PUZZLE 5.2 [44] using HKY substitution model without gamma distribution found by “Findmodel”. Rooting in PUZZLE was done by automatic search for best place.

The demographic history of the vector *H. obsoletus* was evaluated from population samples using D and Fs as demographic indices of the neutrally evolving mtDNA partial gene sequences, and by testing for deviation from a sudden demographic expansion (Arlequin 3.5). Because the outcomes of neutrality and/or demographic tests are influenced by how populations are defined [45] and sampling skew, we implemented the tests with and without *a priori* knowledge of the organisms, and relative to the sampling scheme. *Hyalesthes obsoletus* was considered both as a single population and as three populations based on the distribution of the mtDNA haplotypes “aa” and “bb”, which separate *H. obsoletus* at the Italian-Slovenian karst divide. The three populations were assigned to a western-lineage (France and Switzerland), an eastern-lineage (Germany, Slovenia, Croatia and Austria) population and the putative centre of origin, Italy. In the demographic analysis we assumed that vectors from the contact zone in Switzerland carrying either the “aa” or the “bb” haplotype each belonged to the eastern or western population group.

## Results

### Stolbur tuf-a genetic diversity

For tuf-a stolbur, we obtained one genotype in 61 *stol-11* sequences (741 characters) (accession no. JQ977744), four genotypes in 62 *secY* sequences (829 characters) (JQ977707–10), thirteen genotypes in 54 *vmp1* sequences (1308 characters) (JQ977721–33) and seven genotypes in 41 *stamp* sequences (459 characters) (JQ977713–19). The three genes *secY*, *vmp1* and *stamp* were polymorphic in Italy and Slovenia whereas Swiss and German populations were polymorphic only for *vmp1*. The number of tuf-a *secY*, *vmp1* and *stamp* genotypes in Italy was 3, 10 and 6, compared to 2, 2 and 3 in Slovenia and Croatia, and 1, 2 and 1 in both Switzerland and Germany. Genetic diversity in Italy compared to Slovenia and Croatia was *c*. 20 times higher in *vmp1* (0.00979 vs. 0.00050) and *c*. 10 times higher in *stamp* (0.01028 vs. 0.00117) (Table 3). Genotypes of *vmp1* were shared between Italy, Slovenia and Croatia (genotype N3) and between Germany and Switzerland (N1, N2) (Appendix S3). *Stamp* genotypes were shared between France, Switzerland, Germany and Italy (S1) and between Italy, Slovenia and Croatia (S2, S3). Switzerland and Germany never shared genotypes with Slovenia and Croatia. The frequency distributions in these two geographic regions were highly significantly different, *vmp1*: χ^2^ = 40.00, 3 df, *P*<0.0001; *stamp*: χ^2^ = 27.00, 3 df, *P*<0.0001.

**Table 3:** Genotypic diversity of stolbur genes *secY*, *stamp* and *vmp1* in four predefined European regions, (1) the host-shift population Germany, (2) a putative transition population Switzerland, and the two putative regions of origin, (3) Italy and southern France, and (4) Slovenia and Croatia.

Gene	Region	Number of isolates tested	Number of genotypes	Nucleotide diversity	Mean number of pair-wise differences
*secY*	Germany	13	1	0	0
	Switzerland	17	1	0	0
	Slovenia/Croatia	9	2	0.00027	0.22
	Italy/France*	23	3	0.00325	2.69
*stamp*	Germany	12	1	0	0
	Switzerland	5	1	0	0
	Slovenia/Croatia	10	3	0.00097	0.56
	Italy/France*	14	6	0.01028	5.88
*vmp1*	Germany	13	2	0.00012	0.15
	Switzerland	16	2	0.00010	0.13
	Slovenia/Croatia	11	2	0.00050	0.66
	Italy/France*	14	10	0.00979	12.69

The region Switzerland includes Swiss samples north and west of the Alps. The region Italy/France includes samples from the Swiss canton Ticino, situated south of the Alps and part of the Italian Po Basin. The gene stol-11 was monomorphic in tuf-a stolbur and not included. Genotype frequencies are shown in Appendix S3.

* includes the Swiss canton Ticino south of the Alps.

### Stolbur tuf-a gene phylogenies

**Figure 2:**
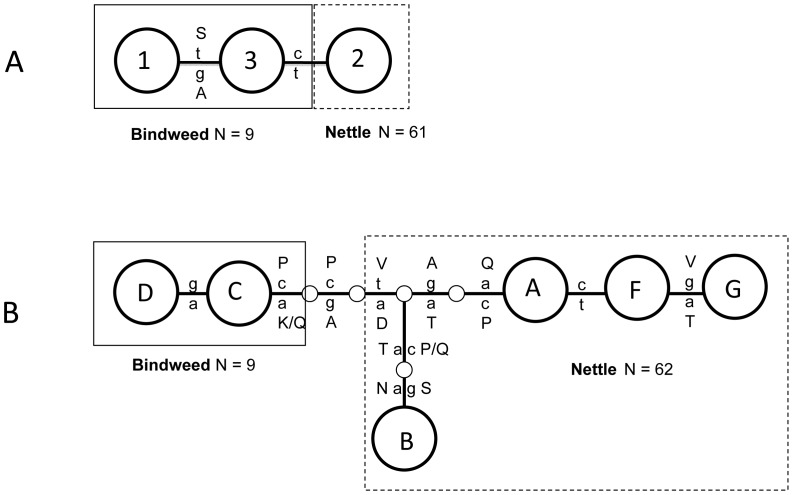
Haplotype networks for genotypes of *stol-11* and *secY*. The networks of *stol-11* (A) and *secY* (B) show all genotypes observed in tuf-a (nettle) associated strains and in nine random, comparative tuf-b (bindweed) strains. Lower case letters: nucleotide substitutions; upper case letters: amino acid substitutions. The corresponding SEE-ERANET nomenclature of the *secY* genotypes is shown in Appendix S3.

Tuf-a genotypes were monophyletic relative to tuf-b genotypes at all four genes (see below) and linked in two clusters corresponding to the two RFLP patterns found in the tuf gene, tuf-a and tuf-b. The tuf-a *stol-11* genotype differed by 1 bp to the most related of the two observed tuf-b genotypes (JQ977745–46) (Fig. 2a). The tuf-a *secY* genotypes differed by minimum four substitutions to the two tuf-b genotypes (JQ977711–12) found in this survey (Fig. 2b).

**Figure 3:**
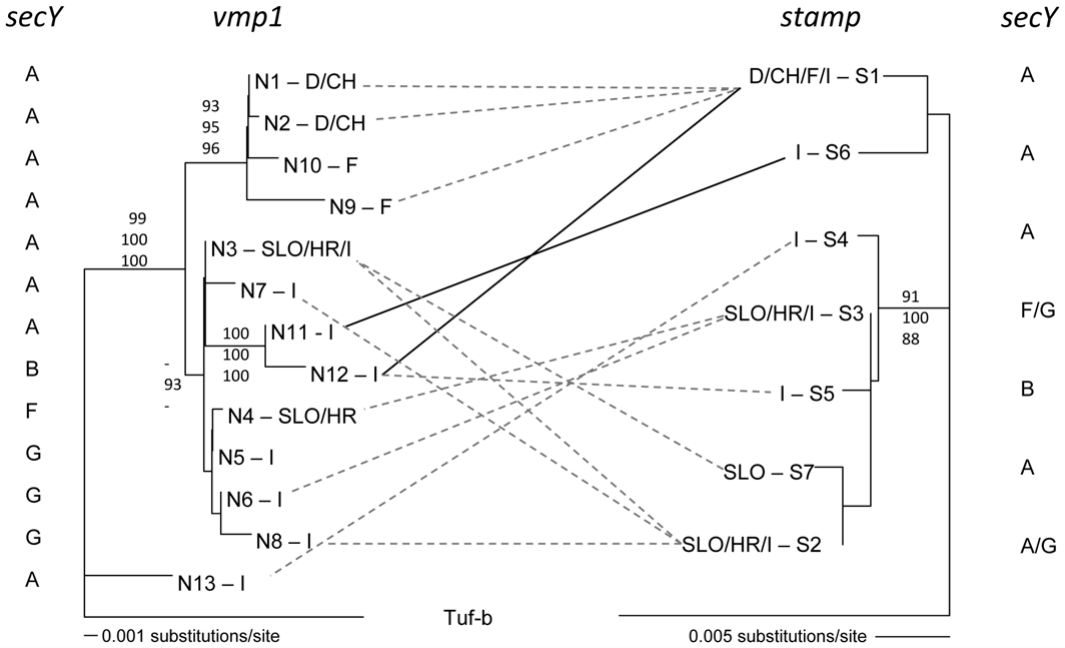
Comparative phylogenetic relationships of stolbur tuf-a *vmp1* and *stamp* genotypes based on isolates sequenced for both genes, and corresponding *secY* genotypes. Bootstrap scores above 80 are given in following order, NJ, MP and ML. Lines show linked genotypes within isolates: congruent cluster = grey hatched line, incongruent cluster = bold full line. The Italian isolate Rome 7 exhibited the genotypes N11 (*vmp1*) and s6 (*stamp*), which belong to different clusters in the two phylogenetic trees (bold full line). Isolates with the *vmp1* genotype N12 had *stamp* genotypes s1 (bold full line) or s5 (hatched line). Note that genotypes in France, Germany and Switzerland north of the Alps cluster together in both trees, and that *secY* genotypes cluster according to the *vmp1* branching pattern. The *secY* genotype A is the ancestral tuf-a genotype. Geographic abbreviations: F = France, I = Italy, HR = Croatia, SLO = Slovenia, D = Germany, CH = Switzerland. The corresponding SEE-ERANET nomenclature of the tuf-a genotypes is given in Appendix S3.

The thirteen tuf-a *vmp1* genotypes were monophyletic relative to the nine paraphyletic tuf-b genotypes (JQ977734–42) observed in this survey (Appendix S4). All observed *vmp1* genotypes exhibited three highly homologous repeated domains of which the first domain in the tuf-a cluster was characterised by a diagnostic penta-peptide (Asp-Val-Ala-Asn-Asn) (Appendix S5). Phylogenetic analyses with ML, NJ and MP produced identical topologies. Fig. 3 shows the phylogenetic relationship of tuf-a *vmp1* with the tuf-b isolate Neuweiher57 as an out-group, ML (−ln = 2311.48036), NJ (ME score = 0.06674); MP analysis found three most parsimonious trees, each with identical main clusters. The MP analysis included 28 parsimony informative and 54 un-informative sites (tree length: 87; retention index: 0.90; consistency index: 0.94; homoplasy index: 0.06). The four tuf-a genotypes found in France, Germany and Switzerland clustered with high bootstrap scores, while genotypes found in Slovenia and Croatia were related (N3) with Italian ones.

The seven tuf-a *stamp* genotypes differed by at least 12 substitutions to the two observed tuf-b genotypes (JQ977720, FN813260.1 [36]). The phylogeny of tuf-a *stamp* corroborated the phylogeny of *vmp1* genotypes in the geographic distribution of related genotypes where genotypes found in France/Germany/Switzerland (s1) and Slovenia/Croatia (s2, s3, s7) belonged to separate clades; ML (−ln = 810.33835), NJ (ME score = 0.04794); MP analysis found two most parsimonious trees, each with identical main clusters. The MP analysis included 5 parsimony informative and 17 un-informative sites (tree length: 45; retention index: 0.94; consistency index: 0.98; homoplasy index: 0.02). Although the bootstrap score separating the genotypes s1 and s6 from the remaining genotypes was slightly below 70, the two genotypes were characterised by a 6 bp deletion/insertion, as described in [36] (Appendix S6).

Multilocus genotypes including the three polymorphic genes were obtained from 33 tuf-a isolates (Appendix S7). Incongruent tuf-a phylogenetic relationships of *vmp1* and *stamp* were observed in isolates with the *vmp1* genotypes N11 and N12. The (single) Italian N11 isolate had the *stamp* genotype s6, which clustered with the western distributed genotype s1. For the genotype N12, the incongruence was more pronounced: N12 isolates from Cembra Valley (Italy) had the western distributed genotype s1 while N12 isolates from Biasca (Ticino, Switzerland) had the eastern distributed genotype s5. Both N12 isolates had the *secY* genotype B.

### Molecular evolution and signals of hybridisation in tuf-a genotypes

Tuf-a *stamp* and *secY* genotypes did not deviate from neutral clock expectations (P>0.10), whereas the 13 tuf-a *vmp1* genotypes did; *vmp1* likelihood ratio test, P<0.001 (log L without clock: −2162.22, independent branch parameters: 22; log L with clock: −2189.02, independent branch parameters: 8; Likelihood ratio test statistic delta: 57.71, df = 14). Fu's Fs was significantly negative across the 13 tuf-a *vmp1* genotypes (Fs = −4.90, P = 0.015) and marginally significant in seven *stamp* genotypes, Fs = −2.52 (Table 4). The ratio of non-synonymous to synonymous sites (D_N_/D_S_) for *secY*, *stamp* and *vmp1* was 4, 3 and 3, respectively. The high non-synonymous to synonymous ratio in *SecY* was caused by non-synonymous substitutions between tuf-a and tuf-b and relative to genotype B, which was intermediate between the two major clades.

**Table 4:** Neutrality tests for *vmp1*, *stamp* and *secY* stolbur phytoplasma tuf-a genes, and demographic tests for the vector *Hyalesthes obsoletus* using the mtDNA genes ND1 and COII.

Species	Gene/Level	N	Tajima's D	Fu's Fs	Demographic expansion, P value	Spatial expansion, P value
Stolbur	*vmp1*	13	−1.27	−4.90*	-	-
	*stamp*	7	−0.49	−2.52+	*-*	-
	*secY*	4	−0.31	−1.16	-	-
*H. obsoletus*	Total population	175	−1.32^x^	−7.44**	*0.006*	*<0.001*
	Italy	42	−1.25	−4.89**	0.12	0.09
	Western Population (F-CH)	59	−1.37^x^	−3.12*	0.44	0.11
	Eastern population (SLO-A-D)	74	−0.15	−0.02	0.23	*0.03*

N = number of sequences (molecular level) or individuals (population level) included in analysis. Note that the populations of *H. obsoletus* were grouped according to mtDNA distributions, F = France, CH = Switzerland, D = Germany, A = Austria, SLO = Slovenia. Significance:

* P<0.05,

** P<0.01,

*** P<0.001. Marginal significance:

x 0.05<P<0.10,

+ P = 0.047, Fu's Fs significant P<0.02. - not applicable.

A comparison of tuf-a and tuf-b *vmp1* sequences suggests that the high number of amino acid substitutions is caused by a combination of insertions and deletions in the repeated domains (Appendix S5, see also [46]). Because *vmp1* is characterised by highly similar repeated domains in both strains, events of hybridisation between strains rather than rearrangements within strains are difficult to verify. Based on the available sequences, there are two putative hybridisation events between tuf-a and tuf-b-strains involving the oligopeptide TPTQDTV. The oligopeptide sequence was found in the second repeat of four type-b genotypes (amino acid position 222–228) in combination with the sequence AGSLTV (position 235–240). Among all tuf-a sequences, this series was observed exclusively in the tuf-a N13 genotype. (It should be noted that the AGSLTV motif alone is uninformative of hybridisation as it is found in all genotypes in both strains.) The TPTQDTV peptide was further observed in the tuf-a genotypes N11 and N12 in the first repeated domain.

Putative signals of recombination in *stamp* were found in tuf-a genotypes s1 and s6, where the amino acid sequence at positions 35–60 was more tuf-b-like (Appendix S6).

Due to the signals of positive selection and/or hybridisation in tuf-a, a demographic analysis was omitted.

### Genetic diversity and demography of H. obsoletus

*Hyalesthes obsoletus* mtDNA haplotype diversity and haplotype distributions of the combined COII/ND1 sequence (1130 bp) in the present study corroborated previous observations [32] (Table 2) showing that the ancestral haplotype “ab” was found throughout southern Western Europe, while “aa” and “bb” occurred east and west, respectively, of the Italian-Slovenia karst divide. Haplotype diversity was higher in Italy than in the western and eastern ranges, number of haplotypes: Italy = 10, western range = 5, eastern range = 3; nucleotide diversity×10^−3^: Italy = 1.004, western range = 0.274, eastern range = 0.314 (Appendix S8). The haplotypes “ab” and “bb” both are centres in star-like networks, whereas “aa” is not (Appendix S9). The frequency of the haplotype “bb” or “bb”-derived haplotypes increased towards its western range limit in France (0.83) and Switzerland (0.85). Haplotype “bb” was found in *H. obsoletus* caught on both stinging nettle and bindweed (Table 2), i.e. there was no evidence for plant associations in mtDNA, thus corroborating the lack of plant associations in the eastern haplotype “aa” [32]. The two northern most Swiss populations, Arlesheim and Hallau, had a mix of “bb” and “aa” haplotypes. Thus, the contact zone between “aa” and “bb” is northern Switzerland and southern Germany.

The analysis of demographic evolution of *H. obsoletus* populations found marginally significant negative D = −1.37 (P = 0.08) and significant negative Fs = −3.12 (P<0.02) for the western population, while deviations from demographic and spatial expansions were not significant (Table 4). The eastern population showed significant deviation from spatial expansion only (P = 0.03). The Italian population exhibited significant negative Fs = −4.89 (P<0.01) but no deviations from sudden expansion models (P>0.09).

The analysis of all individuals from the total sampling area produced a contradictory result: while D was marginally significant (D = −1.32, P = 0.08) and Fs significantly (Fs = −7.44, P<0.01) negative, indicating population expansions, the total population also deviated significantly from sudden demographic and spatial expansion models (P<0.006). The contrasting results are explained by two geographically separated and monomorphic haplotype distributions (“aa” and “bb”) which led to an excess of few steps and a deficit of large steps (mutation differences) in the expected mismatch distribution. The haplotype frequency distributions are shown in Appendix S8.

### Co-dispersal of tuf-a stolbur phytoplasma and H. obsoletus

**Figure 4:**
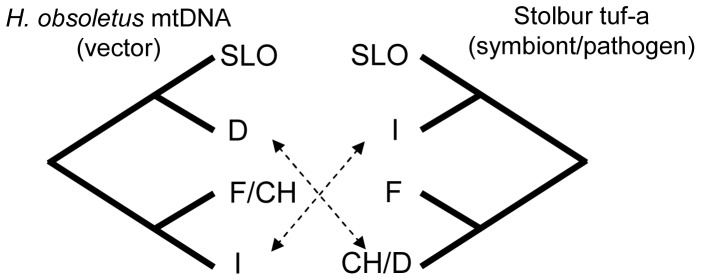
Summary co-dispersal analysis of *H. obsoletus* and tuf-a stolbur based on genealogies and frequency distributions of genetic markers for explaining dissemination of tuf-a in north-western Europe. The figure combines Fig. 1a and e, showing incongruent historical dispersal events and indicates that dissemination of tuf-a in north-western Europe was caused by introduction of tuf-a by secondary immigration of vectors (scenario 3).

The historical co-dispersal of the vector *H. obsoletus* and tuf-a stolbur based on the phylogenies and genetic frequency distributions analysed above is summarised in Fig. 4. The figure combines the hypothesis settings of Fig. 1a (vector) and Fig. 1e (tuf-a stolbur) and shows non-concordant phylogenetic and geographic distributions between tuf-a (based on *vmp1* and *stamp* genotypes) and vector mtDNA haplotypes for populations in North-western Europe (Germany). The vector's haplotype frequency distributions and genealogical relationships presented in Fig.1a and based on [32] were confirmed in the present study. Non-concordance was caused by German vectors of the eastern “aa” lineage, which originated in Slovenia, being infected with the western tuf-a genotype N1s1 found in France and Switzerland.

## Discussion

In this study, we investigated the origin and population history of an emerging insect-vectored plant pathogen by considering how vector dispersal and plant (host) fidelity of both the pathogen and the vector interact on the dissemination process. Specifically, we analysed whether recent dramatic increase in infection pressure on grapevine in the northern, presumably suboptimal range of the vector could be explained by a local host-plant shift of the vector or by vector-mediated immigration of the pathogen. Our results imply that a combination of dispersal (range expansion) and new infections of the vector explain the general dissemination of stolbur tuf-a in Western Europe, and that an introduction of plant-specialised pathogens from plant-unspecialised to plant-specialised vectors during the range expansion explains high infection pressure in the north western range. These results highlight a complex epidemiology of stolbur tuf-a evolving after range expansion from a common source population.

### Co-origins and population expansion

Parallel genetic diversity patterns in vector and tuf-a populations in Western Europe provide strong evidence for a common origin south of the European Alps. The vector was genetically most diverse in Italy where the ancestral haplotypes “ab” was frequent, diversity decreased towards the eastern and western range edges, which did not share the derived signature haplotypes “aa” and “bb”. These diversity patterns were repeated in tuf-a stolbur: genotypes were basal and diversity by far the highest in Italy. In Italy, *vmp1* and/or *stamp* genotypes were shared with eastern (Slovenia/Croatia) and western (France/Germany/Switzerland) populations but not between these eastern and western populations, and diversity decreased towards both the western and the eastern range edges. Reduced genetic diversity in newly colonised regions supports founder effects of invasive populations, as shown in leafhopper-transmitted, invasive pathogen *Xylella fastidiosa* subsp. *fastidiosa* of Pierce's disease of grape in North America [47]. Positive selection in *VMP1* via gene rearrangements [46] or the possibility of hybridisation between stolbur isolates might also question the phylogenetic rigor of the basal position of Italian *vmp1* genotypes, e.g. N13. Despite this quandary, selection is not creating random phylogenetic signals at the geographic level because the genealogical and the geographic associations are correlated, although selection/adaptation may explain the dissemination of certain genotypes in different areas.

Signals of demographic expansion of vector populations detected in the western range and in Italy suggests that the dissemination of tuf-a in Western Europe is linked to a secondary range expansion of the vector with recently acquired tuf-a, which were not co-dispersed in prior migration events. Secondary expansion is implied by recent disease outbreaks related to tuf-a throughout Western Europe (see below), but is most readily seen in the vector's North-western range in Germany and Alsace where resident vector populations have the derived eastern “aa” (“Slovenian”) haplotype but lack tuf-a genotypes found in Slovenia. These distributions combined imply that “aa” evolved in Slovenia and spread to Germany and Alsace *before* tuf-a genotypes co-dispersed with the vector into Slovenia. The dispersal of tuf-a into Slovenia is supported by cohesion in microsatellite genetic variance among Italian and Slovenian vector populations (M. Imo, M. Maixner, J. Johannesen, unpublished data). A complicating issue in this secondary expansion scenario is the fact that the expanding haplotype “bb” is found neither in Slovenia nor in northern Germany. The paradox can be explained by male-biased gene flow or by mating success of local females. Both processes would preserve the original, maternally inherited mtDNA haplotypes. Male-biased gene flow is supported by field observations, which show that males move much more readily between plants than females (M. Maixner and J. Johannesen, unpublished data).

Pathogen dissemination has been linked with range expansions of the vector in other studies [9], [10], [48], [49] while yet others have failed to find associations. For example, the phylogeography of the Lyme disease pathogen, *Borrelia burgdorferi* is not correlated with its tick vectors, *Ixodes scapularis* [50] probably due to independent behaviour (dispersal) of the tick vectors by their avian hosts [51]. The two different outcomes of vector dispersal and pathogen dissemination, uncorrelated and correlated dispersal, are likely influenced by the trophic level of the interactions. However, as we show for *H. obsoletus* carrying tuf-a stolbur phytoplasma, dissemination in the same system may differ if host-specialisations of vector and pathogen are variable.

### Plant fidelity and dissemination

The second part of our study addressed how plant fidelities of the vector and pathogen influence the emergence of tuf-a induced grapevine disease bois noir in North-western Europe (Fig. 4). In corroboration with the vector's range expansion in its western range, we found strong support for immigration of the tuf-a N1s1 genotype from France via Switzerland into Germany, and thus, in the North-western range, a new acquisition of tuf-a that is independent of the vector's host-shift but where the vector's host shift is essential for the dissemination of stolbur tuf-a (scenario 3, Fig. 1e). Monophyly and linkage of tuf-a genotypes at several genes precludes an associated host shift of vector and stolbur in North-western Europe (scenario 1) while at the same time providing support for an independent evolution of tuf-a and –b associated with the two plant complexes. Historical co-migration of unspecialised *H. obsoletus* and stolbur tuf-a from Slovenia (scenario 2) is also rejected because the stolbur tuf-a genotypes east of the Slovenian-Italian karst divide are unrelated to those found in North-western Europe.

The observed genetic pattern, scenario 3, suggests that immigrating plant-unspecialised vectors have transferred tuf-a (genotype N1s1) stolbur to plant-specialised vectors north of the contact zone. An expansion of genotype N1s1 vectored by resident German vectors into southern areas is unlikely because N1 is a tip-haplotype and is not related to Slovenian haplotypes. The complex nature of the vector-tuf-a interaction in the western geographic range is consistent with two general findings observed in host-pathogen systems, but at different interaction levels. First, we detected the emergence of an ecologically specialised pathogen, a phenomenon that might be common in specific host-pathogen associations [52], relative to its natural host plant. Second, we found no evidence that the evolution of plant fidelity of the vector facilitated divergence of tuf-a [53] on the natural host, although the time for genetic imprints thereof might be too short in the current system. Thus, divergence of the two stolbur strains tuf-a and -b primarily follow the natural host plants, while the transmission breadth to agricultural crops is influenced by the natural plant preference of the vector.

These complex interactions are related to the mosaic of co-evolution [54], which predicts different selection and/or historical idiosyncrasies in different geographic regions and thus the potential for divergent evolutionary trajectories in co-evolving systems. For *H. obsoletus* and tuf-a stolbur, we have shown that obligate vector-transmitted tuf-a stolbur are greatly influenced by geographic context in the sense of the vector's plant fidelity.

### Ecology and history of tuf-a dissemination in North-western Europe

The epidemiology of stolbur disease in most agricultural crops is related to the pathogen's distribution in weedy host plants and should therefore be traced in the weeds rather than the crops. The first evidence for stolbur-induced (bois noir) symptoms in grapevine in North-western Europe come from the German Moselle Valley and probably dates back to the 1930's [55], which, perhaps coincidentally, coincides with the first report of the vector in Germany [56]. Bois noir, described at that time was confused with Flavescence dorée [57], remained restricted to a few locations until the late 1990's. Because the tuf-types were not distinguished until 2004 [16], the tuf-type of these first bois noir incidences is unknown but as *H. obsoletus* was observed with field bindweed prior to the present bois noir epidemics, bois noir was most likely caused by tuf-b before 1990.

The late awareness of the presence of two stolbur strains precludes a precise overview of the emergence of the new tuf-a strain before 2004. A survey of the abstract books of the European Bois Noir Workshops [58], [59] and the International Phytoplasmologist Working Group Meetings [60], [61] points towards high relative incidences in Northern Italy and North-western Europe and low relative incidences elsewhere. The dominance of tuf-a in the former region is associated with two observations. First, the emergence is correlated with rising mean temperatures [62], which may have mediated both the observed secondary range expansion into the area as well as survival and specialisation on stinging nettle in previously climatically adverse regions. The second aspect concerns why tuf-a dominates relative to tuf-b in the western compared with the eastern distribution range. Interestingly, the dominance is related to genotype N1s1, which might show traces of hybridisation, a phenomenon that has been linked to the rapid spread of emerging diseases in various organisms, e.g. Dutch Elms disease [63], swine flu [64] and possibly in the planthopper-transmitted Pierce's disease of grape [47]. The phylogenies of *vmp1* and *stamp* genotypes in French, German and Swiss tuf-a isolates indicate traces of both within-tuf-strain (N11-s6 and N12-s1) and between-tuf-strain (stamp: s1 and tuf-b genotypes) hybridisation. The potential for hybridisation between the two tuf-strains is witnessed in the (rare) presence of both strains in stinging nettle as well as in the vector (M. Maixner, unpublished data), and may even occur between mollicute taxa [65]. Because N1s1 is found in stinging nettle populations of the vector that are genetically distinguishable (in Germany) as well as indistinguishable (in Switzerland) (M. Imo, M. Maixner & J. Johannesen, unpublished data) from field bindweed populations, immigration of the genotype into North-western Europe cannot explain host-race divergence of the vector *per se* although association with the genotype might be involved in the vector's ability to exploit the plant. Still, a genome scan of stolbur isolates is required to circumstantiate the hybridisation hypothesis.

The plant specificity of tuf-a raises the question of the vector's original association with stinging nettle. As far as we know, neither stinging nettle nor the vector shows symptoms of tuf-a infection, which suggests long co-evolutionary relationships. For stinging nettle, this is supported by diagnostic mutations in all analysed tuf-a genes [36] (this study). A long relationship between tuf-a and the vector may suggest that stinging nettle previously was a lower ranked host of the vector rather than tuf-a had another vector associated with stinging nettle before *H. obsoletus* acquired it as a new vector, as discussed for the origin of the grapevine yellows disease flavescence dorée (FD) [66]–[68]. For the situation today in North-western Europe where the vector utilises stinging nettle and field bindweed in distinct populations, the introduction of a new symbiont/pathogen has created independent epidemiological cycles resulting in grave disease outbreaks in an accidental foraging-host, grapevine.

## Supporting Information

Appendix S1 **Sampling sites of the stolbur vector** ***H. obsoletus*** **in France, North Switzerland and Germany.** Stolbur isolates were obtained from *H. obsoletus* locations in italics.* Sample sites from [32].
(PPT)

Appendix S2 **Sampling sites of the stolbur vector** ***H. obsoletus*** **in Italy, South Switzerland (Ticino), Slovenia, Croatia and Austria.** Stolbur isolates were obtained from *H. obsoletus* locations in italics.* Sample sites from [32].
(PPT)

Appendix S3 **Genotype frequencies of stolbur tuf-a** ***vmp1*** **,** ***stamp*** **and** ***secY*** **genotypes estimated in four pre-defined European regions, (1) the host-shift population Germany (D), (2) a putative transition population Switzerland (CH), and the two putative regions of origin, (3) Italy and southern France (I/F), and (4) Slovenia and Croatia (SLO/HR).** The region Switzerland includes Swiss samples north and west of the Alps. The region Italy/France includes samples from the Swiss canton Ticino, situated south of the Alps and part of the Italian Po Basin. The gene *stol-11* (accession no. JQ977744) was monomorphic in tuf-a stolbur and is not included. *n* = sample size. Genotype names of the present study are given with the corresponding SEE-ERANET nomenclature in brackets.
(DOC)

Appendix S4 **UPGMA consensus tree (2000 bootstrap replicates) showing monophyly of stolbur tuf-a** ***vmp1*** **genotypes, N1–N13, nested within paraphyletic tuf-b genotypes.** The *vmp1* names in the present study are shown with the corresponding SEE-ERANET nomenclature in brackets.
(PPT)

Appendix S5 ***Vmp1*** **peptide sequence evolution in 13 stolbur tuf-a genotypes associated with stinging nettle and 9 tuf-b genotypes associated with field bindweed observed in this study.** Tuf-a is characterised by a diagnostic penta-peptide at position 165–169. The three repeated VMP1 domains begin at positions 115, 199, 281. Same coloured boxes show origins and putative rearrangement or hybrid motifs. The *vmp1* names in the present study are shown with Genbank accession numbers and the corresponding SEE-ERANET nomenclature in brackets.
(XLS)

Appendix S6 ***Stamp*** **peptide sequence evolution in seven stolbur tuf-a genotypes associated with stinging nettle and two tuf-b genotypes associated with field bindweed observed in this study.** Boxes indicate the putative signal of inter-strain hybridisatin between tuf-a (s1, s6) and tuf-b strains. The *stamp* names in the present study are shown with Genbank accession numbers and the corresponding SEE-ERANET nomenclature in brackets.
(XLS)

Appendix S7 **Multilocus genotypes of 33 stolbur tuf-a isolates for which all three polymorphic genes (** ***secY*** **,** ***vmp1*** **,** ***stamp*** **) were scored.** Genotype names are given for the present study with the corresponding SEE-ERANET nomenclature in brackets.
(DOC)

Appendix S8 ***Hyalesthes obsoletus*** **mtDNA haplotype frequencies in three predefined Western European geographic regions, the putative area of origin (Italy) and the two regions of expansion: West (France, Switzerland) and East (Slovenia, Croatia, Germany) of the European Alps.** The table shows the frequency distributions used in the demographic expansion analysis. The regions were based on findings in [32] and confirmed in the present study, of an eastern and a western historical origin of the haplotypes aa and bb. The frequency distribution in each of the regions East and West are slightly biased towards an overrepresentation of aa or bb, respectively, because animals (haplotypes) in a secondary contact zone between the eastern and western lineages were grouped with the historical region of origin, not the current countries as listed in Table 2.
(DOC)

Appendix S9 **Mitochondrial DNA haplotypic network of the genes COII and ND1 (1311 bp) assayed in Western European** ***Hyalesthes obsoletus*** **.** The haplotype “ab”, connected to the “Pannonian” haplotype “ec”, constitutes the root of the network. The haplotypes “ab” and “bb” are centres of star-like sub-networks, which show signals of demographic expansions in Italy and France/Switzwerland, respectively. The geographic distribution of the haplotypes is presented in Table 2 and Appendix S8.
(PPT)
