# Supplementary material for: Impact of Vector Dispersal and Host-Plant Fidelity on the Dissemination of an Emerging Plant Pathogen
Source: PLoS One. 2012 Dec 19;7(12):e51809. doi: 10.1371/journal.pone.0051809 (PMC3526651; doi:10.1371/journal.pone.0051809)
Supplement: Appendix S1 — Sampling sites of the stolbur vector H. obsoletus in France, North Switzerland and Germany. Stolbur isolates were obtained from H. obsoletus locations in italics.* Sample sites from [32]. (PPT) [file pone.0051809.s001.ppt]

## Slide 1
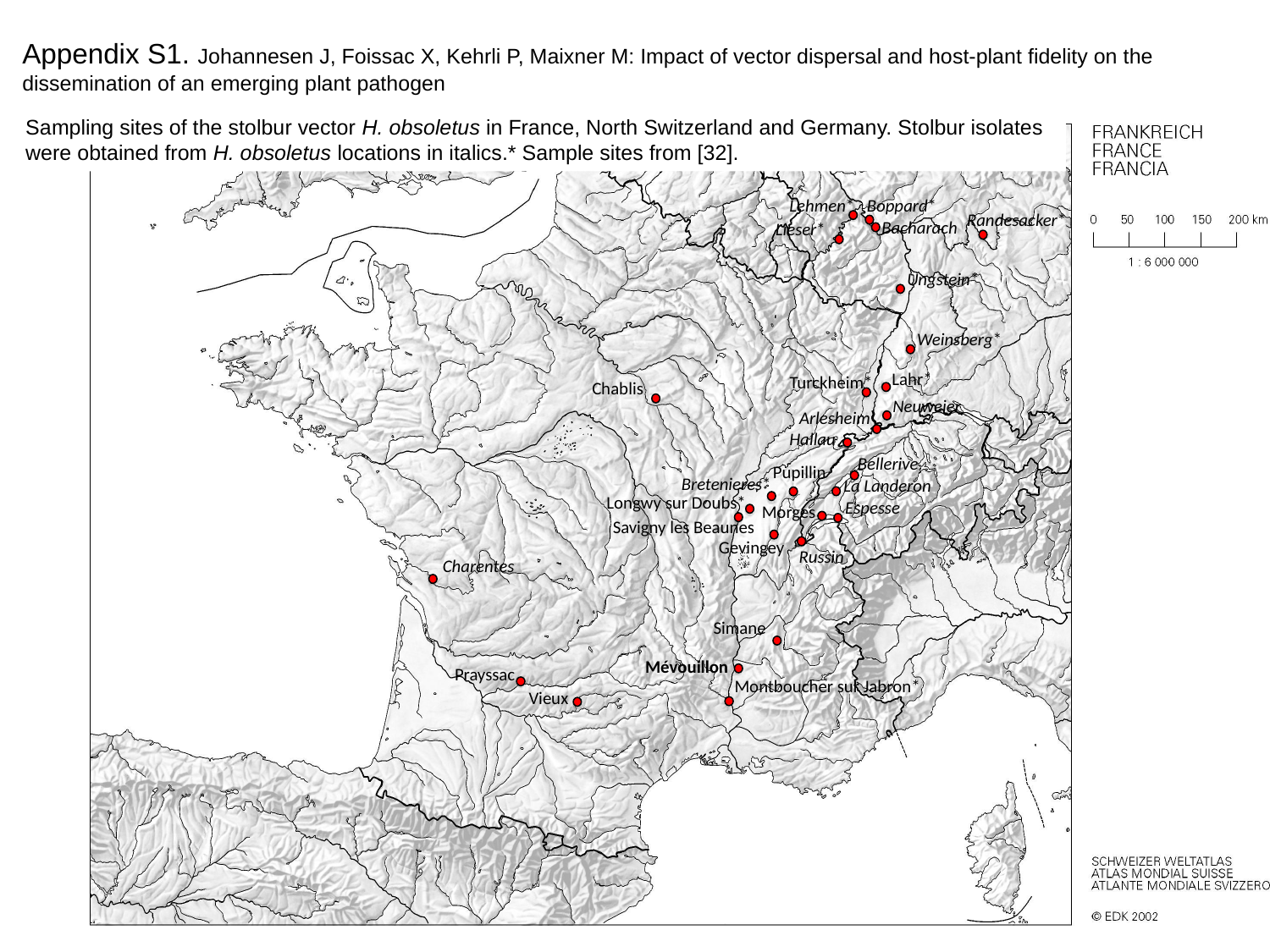

Appendix S1. Johannesen J, Foissac X, Kehrli P, Maixner M: Impact of vector dispersal and host-plant fidelity on the dissemination of an emerging plant pathogen
Sampling sites of the stolbur vector H. obsoletus in France, North Switzerland and Germany. Stolbur isolates were obtained from H. obsoletus locations in italics.* Sample sites from [32].
Lehmen*
Boppard*
Randesacker*
Bacharach
Lieser*
Ungstein*
Weinsberg*
Lahr*
Turckheim*
Chablis
Neuweier
Arlesheim
Hallau
Bellerive
Pupillin
Bretenieres*
La Landeron
Longwy sur Doubs*
Espesse
Morges
Savigny les Beaunes
Gevingey
Russin
Charentes
Simane
Mévouillon
Prayssac
Montboucher sur Jabron*
Vieux
