# Supplementary material for: Impact of Vector Dispersal and Host-Plant Fidelity on the Dissemination of an Emerging Plant Pathogen
Source: PLoS One. 2012 Dec 19;7(12):e51809. doi: 10.1371/journal.pone.0051809 (PMC3526651; doi:10.1371/journal.pone.0051809)
Supplement: Appendix S2 — Sampling sites of the stolbur vector H. obsoletus in Italy, South Switzerland (Ticino), Slovenia, Croatia and Austria. Stolbur isolates were obtained from H. obsoletus locations in italics.* Sample sites from [32]. (PPT) [file pone.0051809.s002.ppt]

## Slide 1
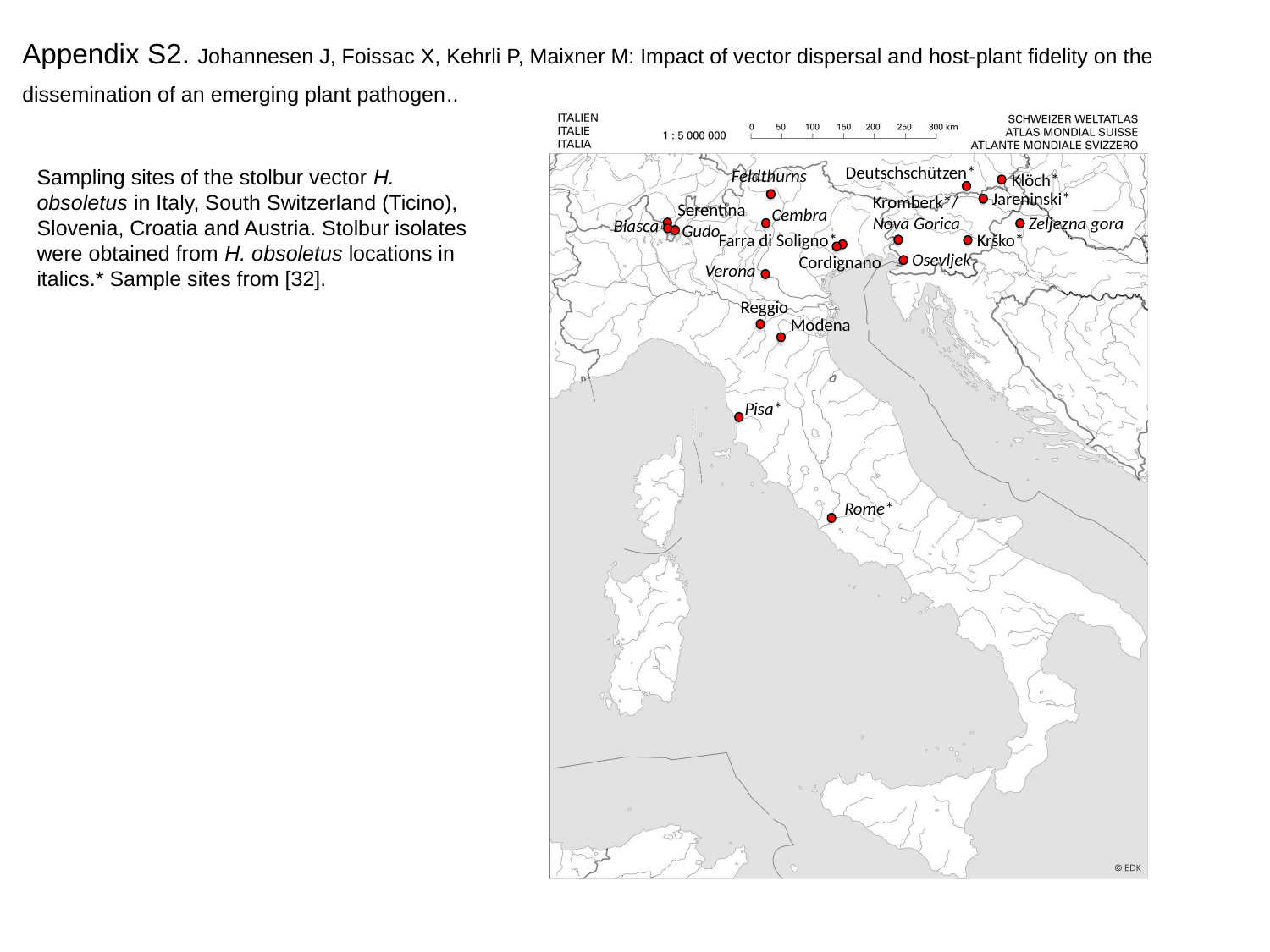

Appendix S2. Johannesen J, Foissac X, Kehrli P, Maixner M: Impact of vector dispersal and host-plant fidelity on the dissemination of an emerging plant pathogen..
Deutschschützen*
Feldthurns
Klöch*
Jareninski*
Kromberk*/
Nova Gorica
Serentina
Cembra
Zeljezna gora
Biasca
Gudo
Farra di Soligno*
Krško*
Osevljek
Cordignano
Verona
Reggio
Modena
Pisa*
Rome*
Sampling sites of the stolbur vector H. obsoletus in Italy, South Switzerland (Ticino), Slovenia, Croatia and Austria. Stolbur isolates were obtained from H. obsoletus locations in italics.* Sample sites from [32].
