# Supplementary material for: Impact of Vector Dispersal and Host-Plant Fidelity on the Dissemination of an Emerging Plant Pathogen
Source: PLoS One. 2012 Dec 19;7(12):e51809. doi: 10.1371/journal.pone.0051809 (PMC3526651; doi:10.1371/journal.pone.0051809)
Supplement: Appendix S3 — Genotype frequencies of stolbur tuf-a vmp1 , stamp and secY genotypes estimated in four pre-defined European regions, (1) the host-shift population Germany (D), (2) a putative transition population Switzerland (CH), and the two putative regions of origin, (3) Italy and southern France (I/F), and (4) Slovenia and Croatia (SLO/HR). The region Switzerland includes Swiss samples north and west of the Alps. The region Italy/France includes samples from the Swiss canton Ticino, situated south of the Alps and part of the Italian Po Basin. The gene stol-11 (accession no. JQ977744) was monomorphic in tuf-a stolbur and is not included. n = sample size. Genotype names of the present study are given with the corresponding SEE-ERANET nomenclature in brackets. (DOC) [file pone.0051809.s003.doc]

**Appendix S3**. Johannesen J, Foissac X, Kehrli P, Maixner M: Impact of vector dispersal and host-plant fidelity on the dissemination of an emerging plant pathogen

Genotype frequencies of stolbur tuf-a *vmp1*, *stamp* and *secY* genotypes estimated in four pre-defined European regions, (1) the host-shift population Germany (D), (2) a putative transition population Switzerland (CH), and the two putative regions of origin, (3) Italy and southern France (I/F), and (4) Slovenia and Croatia (SLO/HR). The region Switzerland includes Swiss samples north and west of the Alps. The region Italy/France includes samples from the Swiss canton Ticino, situated south of the Alps and part of the Italian Po Basin. The gene *stol-11* (accession no. JQ977744) was monomorphic in tuf-a stolbur and is not included. *n* = sample size. Genotype names of the present study are given with the corresponding SEE-ERANET nomenclature in brackets.

|  |  |  | Region |  |  |  |
| --- | --- | --- | --- | --- | --- | --- |
| Gene | Genotype | Accession No. | D | CH | I/F | SLO/HR |
| *vmp1* | N1 (VN1) | JQ977721 | 0.92 | 0.94 | 0 | 0 |
|  | N2 (VN2) | JQ977722 | 0.08 | 0.06 | 0 | 0 |
|  | N3 (VN3) | JQ977723 | 0 | 0 | 0.07 | 0.82 |
|  | N4 (VN4) | JQ977724 | 0 | 0 | 0 | 0.18 |
|  | N5 (VN5) | JQ977725 | 0 | 0 | 0.07 | 0 |
|  | N6 (VN6) | JQ977726 | 0 | 0 | 0.07 | 0 |
|  | N7 (VN7) | JQ977727 | 0 | 0 | 0.07 | 0 |
|  | N8 (VN8) | JQ977728 | 0 | 0 | 0.07 | 0 |
|  | N9 (VN9) | JQ977729 | 0 | 0 | 0.07 | 0 |
|  | N10 (VN10) | JQ977730 | 0 | 0 | 0.07 | 0 |
|  | N11 (VN11) | JQ977731 | 0 | 0 | 0.07 | 0 |
|  | N12 (VN12) | JQ977732 | 0 | 0 | 0.35 | 0 |
|  | N13 (VN13) | JQ977733 | 0 | 0 | 0.07 | 0 |
|  | *n* |  | 13 | 16 | 14 | 11 |
|  |  |  |  |  |  |  |
| *stamp* | s1 (ST6) | JQ977713 | 1.00 | 1.00 | 0.36 | 0 |
|  | s2 (ST23) | JQ977714 | 0 | 0 | 0.29 | 0.60 |
|  | s3 (ST46) | JQ977715 | 0 | 0 | 0.07 | 0.30 |
|  | s4 (ST18) | JQ977716 | 0 | 0 | 0.07 | 0 |
|  | s5 (ST19) | JQ977717 | 0 | 0 | 0.14 | 0 |
|  | s6 | JQ977718 | 0 | 0 | 0.07 | 0 |
|  | s7 | JQ977719 | 0 | 0 | 0 | 0.10 |
|  | *n* |  | 12 | 5 | 14 | 10 |
|  |  |  |  |  |  |  |
| *secY* | A (S6) | JQ977707 | 1.00 | 1.00 | 0.35 | 0.89 |
|  | B (S35) | JQ977708 | 0 | 0 | 0.48 | 0 |
|  | F | JQ977711 | 0 | 0 | 0 | 0.11 |
|  | G (S36) | JQ977712 | 0 | 0 | 0.17 | 0 |
|  | *n* |  | 13 | 17 | 23 | 9 |
