# Supplementary material for: Impact of Vector Dispersal and Host-Plant Fidelity on the Dissemination of an Emerging Plant Pathogen
Source: PLoS One. 2012 Dec 19;7(12):e51809. doi: 10.1371/journal.pone.0051809 (PMC3526651; doi:10.1371/journal.pone.0051809)
Supplement: Appendix S4 — UPGMA consensus tree (2000 bootstrap replicates) showing monophyly of stolbur tuf-a vmp1 genotypes, N1–N13, nested within paraphyletic tuf-b genotypes. The vmp1 names in the present study are shown with the corresponding SEE-ERANET nomenclature in brackets. (PPT) [file pone.0051809.s004.ppt]

## Slide 1
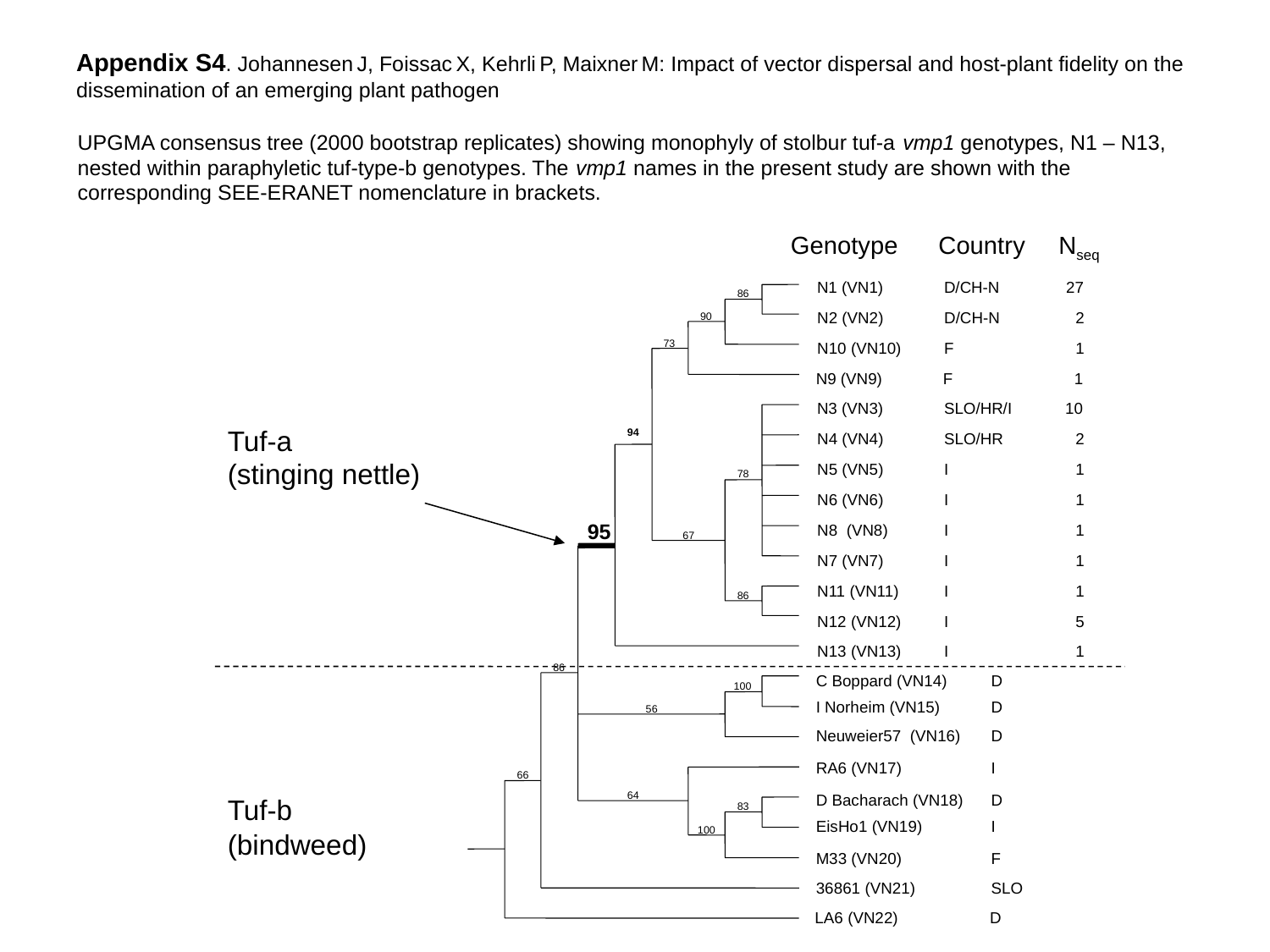

Appendix S4. Johannesen J, Foissac X, Kehrli P, Maixner M: Impact of vector dispersal and host-plant fidelity on the dissemination of an emerging plant pathogen
UPGMA consensus tree (2000 bootstrap replicates) showing monophyly of stolbur tuf-a vmp1 genotypes, N1 – N13, nested within paraphyletic tuf-type-b genotypes. The vmp1 names in the present study are shown with the corresponding SEE-ERANET nomenclature in brackets.
Genotype	 Country	 Nseq
N1 (VN1) 	D/CH-N 27
86
N2 (VN2) 	D/CH-N	 2
90
73
N10 (VN10) 	F	 1
N9 (VN9) 	F	 1
N3 (VN3) 	SLO/HR/I 10
Tuf-a
(stinging nettle)
94
N4 (VN4) 	SLO/HR	 2
N5 (VN5) 	I	 1
78
N6 (VN6) 	I	 1
95
N8 (VN8) 	I	 1
67
N7 (VN7) 	I	 1
N11 (VN11) 	I	 1
86
N12 (VN12) 	I	 5
N13 (VN13) 	I	 1
86
C Boppard (VN14)	D
100
I Norheim (VN15)	D
56
Neuweier57 (VN16)	D
RA6 (VN17)	I
66
Tuf-b
(bindweed)
64
D Bacharach (VN18)	D
83
EisHo1 (VN19)	I
100
M33 (VN20)	F
36861 (VN21)	SLO
LA6 (VN22)	D
