# Supplementary material for: Impact of Vector Dispersal and Host-Plant Fidelity on the Dissemination of an Emerging Plant Pathogen
Source: PLoS One. 2012 Dec 19;7(12):e51809. doi: 10.1371/journal.pone.0051809 (PMC3526651; doi:10.1371/journal.pone.0051809)
Supplement: Appendix S7 — Multilocus genotypes of 33 stolbur tuf-a isolates for which all three polymorphic genes ( secY , vmp1 , stamp ) were scored. Genotype names are given for the present study with the corresponding SEE-ERANET nomenclature in brackets. (DOC) [file pone.0051809.s007.doc]

**Appendix S7**. Johannesen J, Foissac X, Kehrli P, Maixner M: Impact of vector dispersal and host-plant fidelity on the dissemination of an emerging plant pathogen

Multilocus genotypes of 33 stolbur tuf-a isolates for which all three polymorphic genes (*secY*, *vmp1*, *stamp*) were scored. Genotype names are given for the present study with the corresponding SEE-ERANET nomenclature in brackets

| **Abbr./ Isolate** | **Country** | **SecY** | **VMP1** | **Stamp** |
| --- | --- | --- | --- | --- |
| LB2 | D | A (S6) | N2 (VN2) | 1 (ST6) |
| LB-9 | D | A (S6) | N1 (VN1) | 1 (ST6) |
| LB-12 | D | A (S6) | N1 (VN1) | 1 (ST6) |
| RaB7 | D | A (S6) | N1 (VN1) | 1 (ST6) |
| A – Weinsberg | D | A (S6) | N1 (VN1) | 1 (ST6) |
| B – Ungstein | D | A (S6) | N1 (VN1) | 1 (ST6) |
| E – Ungstein | D | A (S6) | N1 (VN1) | 1 (ST6) |
| G – Weinsberg | D | A (S6) | N1 (VN1) | 1 (ST6) |
| H – Bacharach | D | A (S6) | N1 (VN1) | 1 (ST6) |
| N73 | D | A (S6) | N1 (VN1) | 1 (ST6) |
| M | SLO | A (S6) | N3 (VN3) | 2 (ST23) |
| 36817, Ose-SLO-3 | SLO | A (S6) | N3 (VN3) | 2 (ST23) |
| 36819 Ose-SLO-5 | SLO | A (S6) | N3 (VN3) | 2 (ST23) |
| 36825, Ose-SLO-11 | SLO | A (S6) | N3 (VN3) | 2 (ST23) |
| 36826, Ose-SLO-12 | SLO | A (S6) | N3 (VN3) | 2 (ST23) |
| 36832 NG-B2 | SLO | F | N4 (VN4) | 3 (ST46) |
| 36842, NG-B12 | SLO | A (S6) | N3 (VN3) | 7 |
| 36916, ZeG23-HR | HR | A (S6) | N3 (VN3) | 2 (ST23) |
| RB7 | I | A (S6) | N11 (VN11) | 6 |
| RB15 | I | A (S6) | N13 (VN13) | 4 (ST18) |
| 26541 | I | G (S36) | N8 (VN8) | 2 (ST23) |
| Cemb 1,3 | I | B (S35) | N12 (VN1) | 1 (ST6) |
| Cemb 1,7 | I | B (S35) | N12 (VN12) | 1 (ST6) |
| Eis 1,1 | I | A (S6) | N7 (VN7) | 2 (ST23) |
| 28423 | I | A (S6) | N3 (VN3) | 2 (ST23 |
| 28424 | I | G (S36) | N6 (VN6) | 3 (ST46) |
| 28435 | F | A (S6) | N9 (VN9) | 1 (ST6) |
| Bel-5 | CH | A (S6) | N1 (VN1) | 1 (ST6) |
| Lal-10 | CH | A (S6) | N1 (VN1) | 1 (ST6) |
| Rus-8 | CH | A (S6) | N1 (VN1) | 1 (ST6) |
| Hal-7 | CH | A (S6) | N1 (VN1) | 1 (ST6) |
| bim1 | CH/I* | B (S35) | N12 (VN12) | 5 (ST19) |
| biw1 | CH/I* | B (S35) | N12 (VN12) | 5 (ST19) |

* Samples from Swiss canton Ticino, part of the Italian Po basin.

Country: D = Germany, SLO = Slovenia, HR = Croatia, I = Italy, F = France, CH = Switzerland.
