# Supplementary material for: Impact of Vector Dispersal and Host-Plant Fidelity on the Dissemination of an Emerging Plant Pathogen
Source: PLoS One. 2012 Dec 19;7(12):e51809. doi: 10.1371/journal.pone.0051809 (PMC3526651; doi:10.1371/journal.pone.0051809)
Supplement: Appendix S8 — Hyalesthes obsoletus mtDNA haplotype frequencies in three predefined Western European geographic regions, the putative area of origin (Italy) and the two regions of expansion: West (France, Switzerland) and East (Slovenia, Croatia, Germany) of the European Alps. The table shows the frequency distributions used in the demographic expansion analysis. The regions were based on findings in [32] and confirmed in the present study, of an eastern and a western historical origin of the haplotypes aa and bb. The frequency distribution in each of the regions East and West are slightly biased towards an overrepresentation of aa or bb, respectively, because animals (haplotypes) in a secondary contact zone between the eastern and western lineages were grouped with the historical region of origin, not the current countries as listed in Table 2. (DOC) [file pone.0051809.s008.doc]

**Appendix S8.** Johannesen J, Foissac X, Kehrli P, Maixner M: Impact of vector dispersal and host-plant fidelity on the dissemination of an emerging plant pathogen

*Hyalesthes obsoletus* mtDNA haplotype frequencies in three predefined Western European geographic regions, the putative area of origin (Italy) and the two regions of expansion: West (France, Switzerland) and East (Slovenia, Croatia, Germany) of the European Alps. The table shows the frequency distributions used in the demographic expansion analysis. The regions were based on findings in [32] and confirmed in the present study, of an eastern and a western historical origin of the haplotypes aa and bb. The frequency distribution in each of the regions East and West are slightly biased towards an overrepresentation of aa or bb, respectively, because animals (haplotypes) in a secondary contact zone between the eastern and western lineages were grouped with the historical region of origin, not the current countries as listed in Table 2.

----------------------------------------------------------

Haplotype Italy West East

----------------------------------------------------------

aa 0 0 0.784

ab 0.405 0.085 0.203

ad 0.024 0 0

af 0.095 0 0

aj 0.024 0 0

cd 0.024 0 0

bb 0.262 0.831 0

db 0 0.017 0

ib 0.071 0 0

ig 0.024 0 0

kb 0.024 0 0

lb 0 0.017 0

nb 0 0.051 0

ob 0.048 0 0

tb 0 0 0.014

-----------------------------------------------------------

N 42 59 74

-----------------------------------------------------------
