# Supplementary material for: Impact of Vector Dispersal and Host-Plant Fidelity on the Dissemination of an Emerging Plant Pathogen
Source: PLoS One. 2012 Dec 19;7(12):e51809. doi: 10.1371/journal.pone.0051809 (PMC3526651; doi:10.1371/journal.pone.0051809)
Supplement: Appendix S9 — Mitochondrial DNA haplotypic network of the genes COII and ND1 (1311 bp) assayed in Western European Hyalesthes obsoletus . The haplotype “ab”, connected to the “Pannonian” haplotype “ec”, constitutes the root of the network. The haplotypes “ab” and “bb” are centres of star-like sub-networks, which show signals of demographic expansions in Italy and France/Switzwerland, respectively. The geographic distribution of the haplotypes is presented in Table 2 and Appendix S8. (PPT) [file pone.0051809.s009.ppt]

## Slide 1
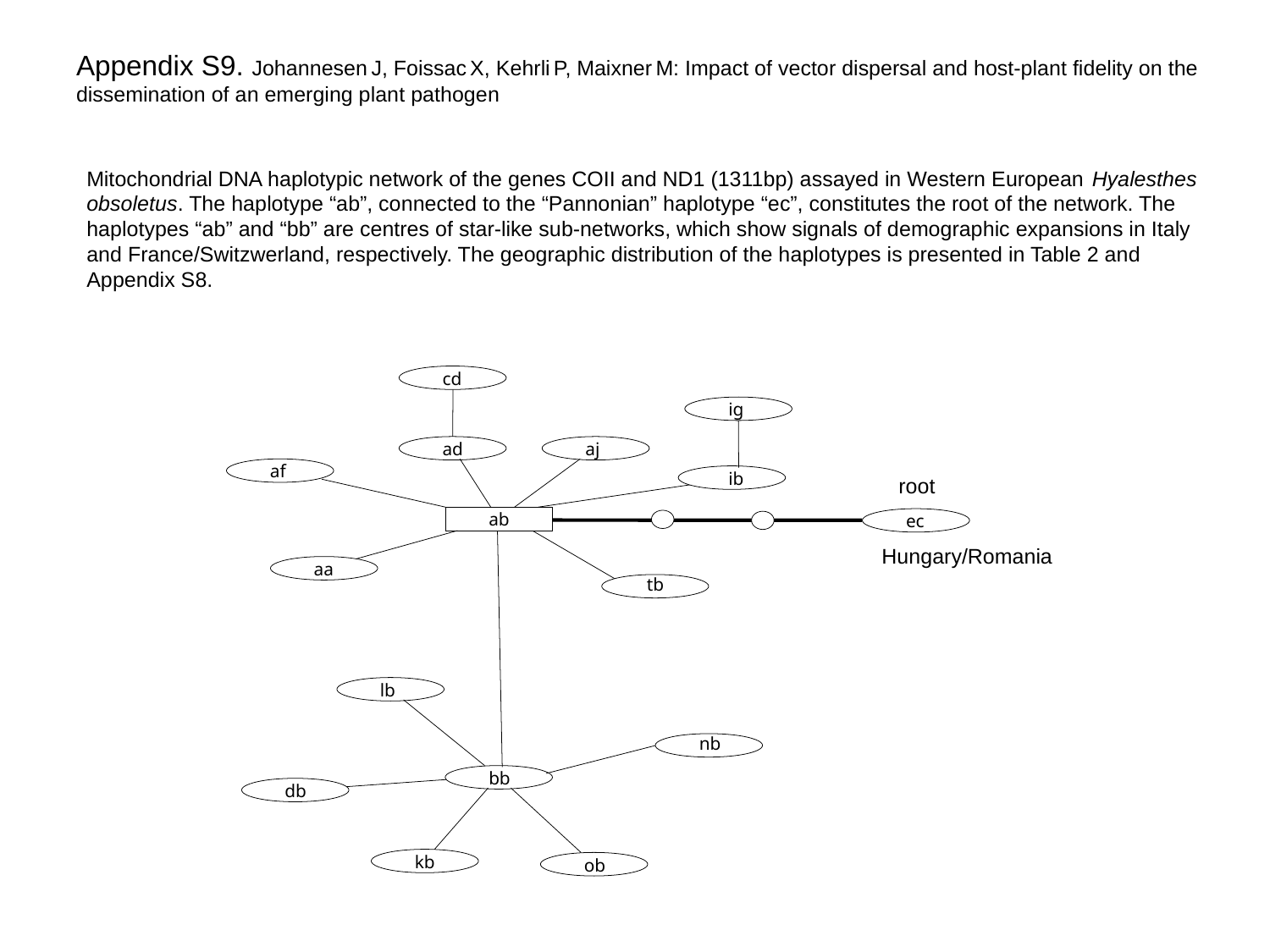

Appendix S9. Johannesen J, Foissac X, Kehrli P, Maixner M: Impact of vector dispersal and host-plant fidelity on the dissemination of an emerging plant pathogen
Mitochondrial DNA haplotypic network of the genes COII and ND1 (1311bp) assayed in Western European Hyalesthes obsoletus. The haplotype “ab”, connected to the “Pannonian” haplotype “ec”, constitutes the root of the network. The haplotypes “ab” and “bb” are centres of star-like sub-networks, which show signals of demographic expansions in Italy and France/Switzwerland, respectively. The geographic distribution of the haplotypes is presented in Table 2 and Appendix S8.
cd
ig
ad
aj
af
root
ib
ab
ec
Hungary/Romania
aa
tb
lb
nb
bb
db
kb
ob
